# The feasibility of team care for women seeking to plan a vaginal breech birth (OptiBreech 1): an observational implementation feasibility study in preparation for a pilot trial

**DOI:** 10.1186/s40814-023-01299-x

**Published:** 2023-05-12

**Authors:** Shawn Walker, Emma Spillane, Kate Stringer, Amy Meadowcroft, Tisha Dasgupta, Siân M. Davies, Jane Sandall, Andrew Shennan, the OptiBreech Collaborative, Avni Batish, Louisa Davidson, Sabrina Das, Lenka Magurova, George Haroun, Charlotte Meates, Gillian Houghton, Helen Le Grys

**Affiliations:** 1 Department of Women & Children’s Health, Faculty of Life Sciences & Medicine, School of Life Course and Population Sciences, King’s College London, London, SE1 7EH UK; 2 Women’s and Children’s Services, Chelsea and Westminster Hospital NHS Foundation Trust, 369 Fulham Rd, London, SW10 9NH UK; 3 Kingston Hospital NHS Foundation Trust, Galsworthy Road, Surrey Kingston upon Thames, KT2 7QB UK; 4 Surrey and Sussex Healthcare NHS Trust, East Surrey Hospital, Canada Avenue, Redhill, RH1 5RH UK; 5 Northern Care Alliance NHS Foundation Trust, Royal Oldham Hospital, Rochdale Road, Oldham, Greater Manchester OL1 2JH UK

**Keywords:** Feasibility, Implementation, Breech presentation, Intrapartum care

## Abstract

**Background:**

OptiBreech Care is a care pathway for breech presentation at term, including where chosen, physiological breech birth attended by professionals with advanced training and/or proficiency. We aimed to assess the feasibility of implementing OptiBreech team care prior to proceeding with a planned pilot randomised controlled trial.

**Methods:**

Our design was an observational implementation feasibility assessment across England and Wales, January 2021–June 2022. Our objectives were to determine whether Trusts could provide attendants with advanced training (implementation feasibility), who deliver protocol-consistent care (fidelity), within existing resources (costs), while maintaining low neonatal admission rates (safety) and adequate recruitment rates (trial feasibility). Participants included women > 37 weeks pregnant with a breech-presenting foetus, requesting support for a vaginal breech birth following standard counselling, and staff involved in the study. No randomisation occurred in this first stage of feasibility work.

**Results:**

Thirteen National Health Service sites were recruited. A total of 82 women planned births in the study. Sites with a breech specialist midwife recruited at double the rate of sites without (0.90/month, 95% *CI* 0.64–1.16 vs 0.40, 95% *CI* 0.12–0.68). Referrals into the study came from midwives (46%), obstetricians (34%) and women themselves (20%). Vaginal births were attended by staff with OptiBreech training at 87.5% (35/40, 95% *CI* 0.732–0.958) and by staff who met additional proficiency criteria at 67.5% (27/40, 95% *CI* 0.509–0.814). Fidelity criteria were more consistently met by staff who also met proficiency criteria. There were four neonatal admissions (4.9%, 4/82), including one serious adverse outcome (1.2%, 1/82).

**Conclusions:**

A prospective observational cohort of OptiBreech collaborative care, which could potentially support nested or cluster randomisation, appears feasible in sites willing to establish a dedicated clinic and strategically develop further proficient members of staff, with back-up plans for supporting rapidly progressing births. Randomisation procedures remain to be feasibility tested. It is funded by the NIHR (NIHR300582).

## Key messages

- Even in sites with minimal experience levels, NHS staff with enhanced training can attend most planned breech births if supported to work flexibly.
- An observational cohort study of OptiBreech collaborative care appears feasible. The feasibility of randomising women to OptiBreech or standard care within the cohort needs to be tested.
- A substantive study is likely to be most successful and efficient in sites with a dedicated clinic supported by a breech specialist midwife and lead obstetrician.

## Background

Breech presentation, where the baby is positioned head up instead of head down, occurs in 4% (1:25) of term pregnancies [1]. A lack of high-quality, recent evidence undermines shared decision-making for women in these pregnancies. Over 96% of all persistent breech babies are born by caesarean in the United Kingdom (UK) [2], and breech is the indication for 14% of all caesareans in countries with a low perinatal mortality rate [3]. The majority of breech presentations occur in first pregnancies, contributing significantly to the most common indication for surgical delivery: previous caesarean [3]. To reduce the caesarean rate for breech, and associated complications, most women are recommended an attempt at external cephalic version (ECV) [4]. An ECV is a procedure to manually turn the foetus head down using pressure on the maternal abdomen [4]. However, while ECV reduces the caesarean birth rate, it has not been shown to improve outcomes for babies, compared to no ECV [5].

The Royal College of Obstetricians and Gynaecologists (RCOG) summary of evidence suggests that with skilled and experienced practitioners, breech birth may be ‘nearly as safe as cephalic [head-down] birth’ (perinatal mortality per 1000: caesarean = 0.5, cephalic birth = 1, breech birth = 2) [1]. Yet across the UK and internationally, women who wish to plan a vaginal breech birth (VBB) have raised concerns about a lack of support [6–9]. While many women wish to plan a caesarean birth, some women report no option but to deliver by caesarean, causing ‘stress, anger, fear and injustice.’ [7] [10] Some feel pressured to attempt an ECV [10–12], and some experience the procedure as very painful, with over 10% describing it as ‘intolerable’ [13].

As many as 25–58% of women may prefer to plan a VBB, but this is highly dependent on the type of counselling they receive [14–16]. Some hospitals have created breech clinics and/or an on-call team to revive breech skills [17, 18], and these attract women who lack local support [19, 20]. The VBB rate can rise as high as 6–11% of the total birth rate due to women travelling to experienced providers [19, 20], compared to 0.4% of the total birth rate in the UK [2]. When one hospital in Belgium implemented specialist team care for physiological breech births, this resulted in a statistically significant increase in vaginal breech births (4.3% to 43.5%; *OR* 17.0; 95% *CI* 7.3–39.6), together with a non-statistically significant decrease in neonatal admissions [21].

Physiological breech birth is an approach to managing VBBs based on evidence about the normal physiology of breech labour and birth and how this is optimised [22–25]. These methods include guidance on indications for intervention based on video and clinical records analysis [23, 24] and use of upright active maternal birth positions [19]. Experienced team care, upright maternal positioning and time-based indications for manual assistance are significant departures from methods of breech delivery that have been used in the UK National Health Service (NHS) and tested in previous trials [26]. Such substantive differences are likely to impact outcomes. To evaluate this impact, we developed a complex intervention that could be tested in a trial, OptiBreech collaborative care (funded by the National Institute for Health and Care Research, NIHR300582).

### Aims and objectives

The overall aim of the OptiBreech project [27] was to determine if it would be feasible to evaluate the outcomes of OptiBreech collaborative care compared to standard care for women with a breech-presenting baby in a trial. Funding was obtained for a pilot trial [28], but potential sites expressed doubt that they would be able to implement the physiological breech birth teams required to conduct the pilot trial, especially during labour and birth. This included the original NHS cosponsor of the study, who withdrew from participation at the start of the COVID-19 pandemic (July 2020). Following discussion with sites about their concerns, it was collaboratively decided with them that a first-stage observational study to evaluate implementation feasibility would help determine whether to proceed with a pilot randomised trial.

The aim of this study (OptiBreech 1) was to assess the feasibility of implementing OptiBreech team care for planned physiological breech births, to determine whether to proceed with a pilot trial. Our objectives were to determine whether NHS maternity services could provide attendants with OptiBreech training (implementation feasibility), who deliver protocol-consistent care (fidelity), within existing resources (costs), while maintaining low neonatal admission rates (safety) and adequate recruitment rates (trial feasibility) Tables 1 and 2.

**Table 1:** Inclusion and exclusion criteria

Inclusion criteria for OptiBreech 1 included the following: •Singleton pregnancy ≥ 37^+0^ weeks with a breech-presenting foetus confirmed by ultrasound scan (for counselling) •Eligible for approach and recruitment from 36^+0^ •Referred by obstetric consultant, midwife or self (from a different hospital) •Requesting a vaginal breech birth under current Trust guideline •Giving informed consent to participate in outcome monitoring as part of the study
Exclusion criteria included the following: •Absolute contraindication to vaginal birth, such as placenta praevia •Multiple gestation (twins, triplets, etc.) •The proficient team declines to offer care because the clinical case is outside the team’s local guideline or experience level (at the discretion of the breech leads)

**Table 2:** Outcomes assessed in the OptiBreech 1 observational study

1.Trial feasibility a.Number of sites that opened and recruited as planned b.Source of participant referral c.Mean and standard deviation recruitment per month across all sites d.Attrition, defined as women who requested a planned caesarean birth following enrolment
2.Implementation feasibility a.Proportion of vaginal breech births attended by a professional who had completed OptiBreech training b.Proportion attended by a professional who met the full proficiency criteria
3.Fidelity — Care provided according to OptiBreech training [24, 36] a.Proportion of vaginal breech births occurring with upright maternal positioning b.Encouragement of maternal movement and effort prior to hands-on assistance c.Less than 5 min between birth of foetal pelvis and birth of the aftercoming head d.Less than 7 min between ‘rumping’ (anus and both buttocks visible) and birth of the aftercoming head e.Initiation of resuscitation (if required) with umbilical cord intact
4.Costs a.Time spent on-call to deliver service b.Cost to achieve full proficiency criteria, assuming no prior experience
5.Safety a.Proportion of neonates admitted to higher-level care immediately following birth b.Combined perinatal mortality or severe morbidity c.Severe unexpected adverse events
6.Effectiveness a.Mode of birth b.Diagnosis prior to or during labour c.Attempt at external cephalic version prior to planned VBB d.Reasons for caesarean birth

## Methods

### Study design

OptiBreech 1 study was a prospective observational study of OptiBreech team care for planned physiological breech births. A detailed implementation process evaluation within feasibility work increases the chances of a successful trial [29]. Implementation outcomes were informed by Proctor et al. and focused on feasibility, fidelity and costs [30]. Acceptability to women and staff was also assessed qualitatively; this is reported separately [31].

#### Patient and public involvement and engagement

The OptiBreech Patient and Public Involvement and Engagement (PPIE) Group is composed of women who have planned a VBB, either within the study or outside of it, and service user representatives who work with this population. We held one face-to-face PPIE meeting during the design phase of the study, including consent procedures [32], and four PPIE meetings during the study via Microsoft Teams. Our early aim was to enable stakeholders to influence the design of the innovation pathway and the study. Later, we aimed to share emerging results and enable stakeholders to influence the way these were interpreted and reported. Participants also requested a closed FaceBook OptiBreech PPIE group, and this was created.

During each of the PPIE meetings, participants raised concerns about the difficulties they experienced accessing support for VBB. This included finding information about how to access OptiBreech care through participation in the study. Participants asked the research team to promote the study more on social media and by contacting maternity services in locations surrounding the OptiBreech sites, and this was done.

We also involved two women with experience of planning a breech birth as co-researchers. In addition to influencing the design of the research, they were particularly involved in helping to interpret results and how they were reported. Another service user representative served on the study steering committee (SSC). These women’s experiences included uncomplicated birth, complicated birth including neonatal death and in-labour caesarean with postnatal complications.

#### Oversight: study steering committee

As this was an observational study, an SSC was appointed. The SSC was chaired by a professor of midwifery and included two independent obstetric representatives with experience of clinical trials and breech research. Members of the research team were also included on the SSC, along with the service user representative. The primary purpose of the SSC was to monitor the progress of the study and conduct and advise on its scientific credibility. Four meetings were held. These included a review of a serious adverse event (SAE) and subsequent protocol changes (August 2021), the decision to proceed to the pilot of randomisation within the four leading sites (November 2021) and early closure of the OptiBreech 1 study (June 2021). A final meeting was held in September 2022 to affirm the plan to continue recruiting participants to the observational study, while further funding was sought.

#### Participants

Recruitment occurred between February 2021 and June 2022, with follow-up until August 2022.

#### Women and pregnant people

Participants who expressed an intention to plan a breech birth under the participating hospital’s current guideline were referred to the local principal investigator (PI) and provided information about the study. All participants were informed that sites were attempting to implement team care to support breech births, but that this could not be absolutely guaranteed. Written consent was obtained, using either in-person or e-consent procedures.

Contact information for PIs was available on the study’s website [33]. Participants who self-referred onto the study from other hospitals did so by contacting one of the PIs or the chief investigator for referral to their nearest OptiBreech site. Where breech presentations were diagnosed in labour and the attending clinicians called an OptiBreech team member for support, consent to contribute data to the study was sought after the birth rather than during labour, in keeping with current RCOG guidance on ‘Obtaining Valid Consent to Participate in Perinatal Research Where Consent is Time Critical’ [34]. Women who were asked for consent were also able to discuss their birth experience with members of their care team, as recommended by our PPIE group.

A modest minimum sample size of 20 planned VBBs was prespecified, as this study was designed to select sites for the pilot randomised trial, and we were uncertain of demand for VBB.

#### Staff

Thirteen NHS hospitals in England and Wales participated. Sites included the following:

- Birmingham Women’s Hospital
- Chelsea and Westminster Hospital, London
- Kingston Hospital, London
- Imperial College London, Queen Charlotte’s and Chelsea Hospital
- Lewisham Hospital, London
- Liverpool Women’s Hospital
- Musgrove Park Hospital, Somerset
- Prince Charles Hospital, Merthyr Tydfil, Wales
- Royal Cornwall Hospital
- Royal Oldham Hospital, Manchester
- Royal Sussex County Hospital, Brighton
- Surrey and Sussex Hospital, Redhill
- West Middlesex Hospital, London

Each site was requested to identify a breech lead obstetrician and a breech lead midwife, one of whom was the PI. The leads were responsible for maintaining records of who had completed OptiBreech training and/or met proficiency criteria to plan intrapartum care. The protocol suggested appointing a ‘breech team’ of five obstetricians and five midwives, and funding was set aside to support their training. However, the research team did not specify the exact arrangements and instead aimed to observe the process individual sites adopted to meet the needs of the service in their contexts.

To support teams through this process, monthly OptiBreech meetings were held. These began with a focus on an aspect of clinical care and ended with an opportunity for reflection and support. ‘Discussions’ described below refer to these numerous informal discussions that took place throughout the study, as well as direct conversations with the PIs. The research team used these discussions to understand how the study processes were working, particularly in cases where the fidelity criteria were not met or a neonatal admission occurred. Notes from these discussions were included in reports to the SSC. Formal interviews conducted with participants concerning the acceptability of team care for VBBs and the methods for qualitative data analysis will be reported separately [31].

#### Innovation

The OptiBreech collaborative care pathway was developed from previous research. Its programme theory is briefly summarised as follows:

- Providing reliable, experienced support for physiological breech births, in which women are encouraged to remain active and adopt the birthing position of their choice (*the intervention*), [24] will improve access to and outcomes of breech births [35].
- Because this is more acceptable to women than standard care, in which skill levels are unpredictably variable and low overall (*mechanism 1*) [36, 37]
- And because birthing in upright positions results in shorter labours and fewer interventions, compared to the supine birthing positions prescribed in standard care (*mechanism 2*) [19, 22]
- But these potential benefits may only be realised in contexts where specialist multidisciplinary teams are enabled to self-organise to support the wider maternity care team as required (*context*) [38, 39].

**Fig. 1:**
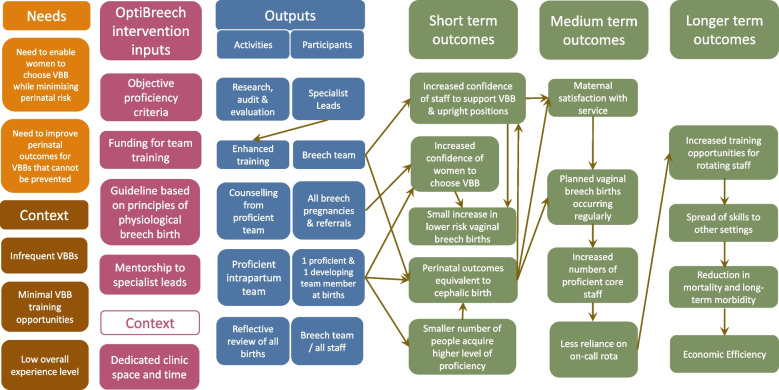
Original OptiBreech logic model, 11 January 2021

**Fig. 2:**
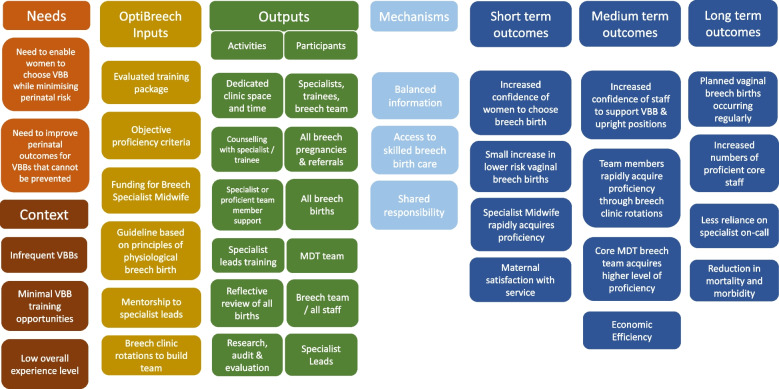
OptiBreech logic model, refined following implementation process evaluation, 4 July 2022

Figure 1 presents the original OptiBreech logic model. Figure 2 presents the current OptiBreech logic model, refined through this early implementation feasibility assessment.

OptiBreech physiological breech birth training was provided free to participating sites by Breech Birth Network, CIC. The training programme had previously been fully evaluated within NHS settings [36]. However, during the COVID-19 pandemic, it became difficult to release staff for in-person training. The entire training package was moved on-line and supplemented with hands-on skills training delivered by leads whenever possible. The proficiency criteria were based on previous research concerning *Standards for maternity care professionals attending planned upright breech births* [39], *Deliberate acquisition of competence in upright breech birth* [38] and *Expertise in physiological breech birth* [35]. These included the following:

- Completion of the OptiBreech training package [36, 37]
- Attendance of at least 10 VBBs including complications [39]
- Attendance of 3 VBBs within the past year [39]
- Contributing to clinical teaching [35, 38]
- Reflective reviews of births attended [35, 38]
- Self-assessment as ready for autonomous practice

A copy of the Physiological Breech Birth Algorithm [23, 24] and a pro forma for documenting critical information around the time of birth were provided to support implementation of the OptiBreech training that all team members should have undertaken. Later, following review of a serious adverse outcome by the SSC, an algorithm to guide decision-making in the second stage of labour was also provided.

#### Outcomes

Primary outcomes assessed were related to trial feasibility, implementation feasibility, fidelity and costs. Secondary outcomes were related to safety and effectiveness.

#### Data collection and analysis

Birth outcome data were collected on paper-based case report forms (CRF), and a copy was sent to the research team by secure encrypted e-mail, along with a copy of the birth pro forma to confirm records relating to the birth time intervals. All data were collected onto a database, and this was double-checked for accuracy by a separate member of the research team against the original CRFs.

Data were analysed using IBM SPSS Statistics (Version 28) software. Descriptive statistics were reported as exact figures and percentages. Confidence interval (CI) for recruitment was calculated using a one-sided t-test for the overall mean. Difference in effect size dependent on different models of care was compared using a one-sided ANOVA. The CI for rates of attendance by clinicians with enhanced training or full proficiency were calculated using the Clopper-Pearson interval test.

#### Changes to study design

An important change to the study design occurred during the set-up phase. The original implementation feasibility objective was to determine only how often births were attended by someone who met the full proficiency criteria, with a 90% target to participate in the pilot randomised trial. However, several sites indicated that this would be difficult or impossible, due to the low overall experience levels they began with. We concluded that if OptiBreech collaborative care was tested in a substantive study or scaled up after a positive pilot trial, many sites would need to pass through a period of building up capacity to meet the ‘proficient attendant’ criteria. Therefore, we decided to set a realistic target for the sites who needed to develop proficient attendants. The revised criteria was ‘attendance by someone who had completed the enhanced OptiBreech training’, at all actual VBBs, with a 90% target, to proceed to the pilot randomisation stage. We continued to also assess the rate of attendance by someone meeting the full criteria.

Additionally, one of the objectives stated in the protocol was to assess how much time it takes to develop a proficient team to deliver the service. Assessing this in terms of ‘time’ was an ineffective approach, affected by too many variables, including staffing levels during the COVID-19 pandemic and baseline physiological breech birth rates within the individual setting. A more useful approach, based on a key implementation outcome [30], is to assess this based on cost. The cost of developing a proficient team member was possible to assess based on the data we collected in this study, including amount of time spent on call to support the births. We have therefore added ‘costs’ above as a secondary objective. Rather than reporting ‘time’ to develop proficient team members, as per our original objective, we report the ‘cost’ of the time needed to develop a proficient team member to deliver the service. Properly costing this time will support replication of the implementation activities if the intervention is widely adopted.

A final change included early closure of the study. OptiBreech 1 was originally scheduled to run until 31 August 2023. In April 2022, the NIHR went through a process of clearing the portfolio of studies following COVID-19, during which many research studies stagnated, with poor recruitment. At this point, the OptiBreech 1 study had over-recruited from our initial target of 40, with 82 women and 21 staff having participated in the study already. At a special meeting of the SSC, it was decided that sufficient recruitment had been achieved to decide on feasibility of a pilot trial and/or continued observational cohort. The OptiBreech 1 study was closed to recruitment in June 2022, and sites remaining open at that time were invited to contribute to the cohort arm of the on-going OptiBreech Care Trial.

## Results

### Trial feasibility

#### Sites

Thirteen sites opened and recruited as planned. Three sites opened but did not recruit to the study. Three sites opened, recruited and then closed early. Thirteen sites listed on our IRAS submission did not open at all.

The original plan was to include 4–5 sites in a pilot trial, following prior testing of implementation feasibility. During the COVID-19 pandemic, it was clear that delivering clinical research would require extraordinary effort from those leading the study locally. The research team therefore welcomed all sites that expressed an interest in trying, and 29 sites were included on the protocol. This also enabled the research team to observe site-specific conditions that resulted in more efficient site opening, higher recruitment figures, higher achievement of proficient attendants and better feedback from women using the service, to inform future site-selection strategy.

The chief investigator formally wrote to each site that opened but did not recruit and asked PIs and Research and Development (R&D) Departments to provide their views on why recruitment did not occur. One of these sites was not able to support planned VBBs in the study due to reported ‘strong resistance from the obstetric clinical lead’ and other members of the obstetric team, despite having an obstetric PI and members of the senior midwifery team committed to being on-call for the births. Another site has a dedicated breech clinic staffed by an obstetrician who completed the training and regularly attends VBBs, but there was no breech lead midwife with capacity to conduct the necessary research activities. The R&D Department was also severely short-staffed, delaying confirmation of capability and capacity for over a year, until just before the OptiBreech 1 study closed. The third did not provide a reason.

Among the three sites that opened and then closed, one reported being unable to release staff for training or to be on call for breech births, due to severe staff shortages. The other two experienced neonatal admissions that may have potentially been associated with protocol violations; following discussion with the SSC, the sites were requested to pause recruitment until further in-person training could be provided to the team. Pandemic conditions prevented this being organised until after the study closed. Key team members at one of the sites have now completed this training, and the site has rejoined the observational arm of the OptiBreech Care Trial.

Among the thirteen sites listed on our IRAS submission that did not open at all, the reasons provided were as follows:

- Insufficient capacity within the R&D Department to open the study (4) — including clinical teams not being able to release research midwives on secondment
- Objections from obstetric or midwifery leadership to participation in the study (4)
- Unable to release staff for training (4)
- Staffing issues prevented enabling people to be on call for breech births (4).
- Insufficient workforce capacity within clinical team to deliver the study (4) — including no PI

A total of three consultant midwives (NHS Band 8) reported unprompted that they left clinical work at the hospital where they were based, in part due to resistance from the obstetric leadership, which prevented the study from opening or breech births from happening without conflict once the study had opened.

#### Principal investigators

Of the thirteen sites that opened and recruited to the study, ten were led by midwife PIs (Table 3). Interactions with R&D offices indicated this was unusual, with some questioning whether it was possible for a midwife to be a PI on a research study, believing it was a requirement to have an obstetric PI. Enabling midwife PIs has been central to our ability to deliver this research.

**Table 3:** Principal investigators

**Profession of PI**	**Total** 29	**Opened & recruited**	**Study site did not open/did not recruit/withdrawn**
Midwife	19^a^	10	10^a^
Obstetrician	9	3	6
Not named	1	n/a	1

^a^One site did open and recruit and then withdrew. Some services had multiple sites, but only one official PI is listed on the IRAS form

All PIs in the sites that opened and remained open were also PIs for the first time. This involved an investment in time and capacity building, requiring extra support from the central research team. Many of the PIs also took responsibility for attending births in the study to support the team members to develop or to support members of the regular clinical team. Discussions with PIs indicated this may be more challenging for obstetric staff, whereas midwifery staff often worked more flexibly, including through on-call arrangements.

One PI stepped down from the role following a severe unexpected adverse outcome, which also had a negative effect on this person’s well-being, as it did for the family involved.

#### Referrals

**Fig. 3:**
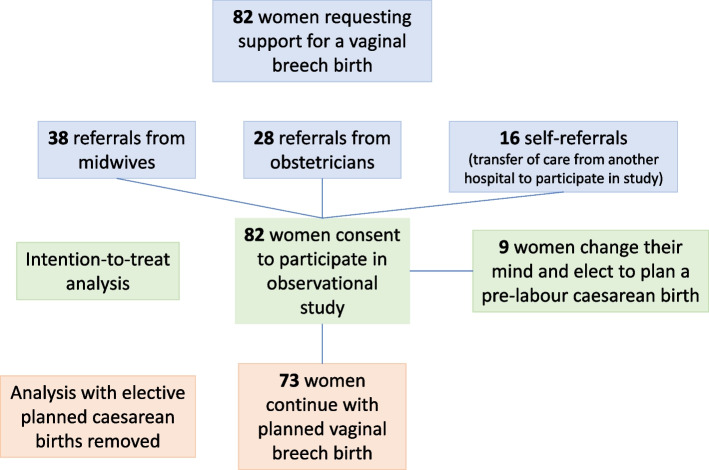
Flow diagram

Referrals to the study came from midwives, obstetricians and self-referrals by the women themselves, via contact information on the OptiBreech website (Table 4, Fig. 3 flow diagram).

**Table 4:** Sources of referral

Midwife	38/82	46.3%
Obstetrician	28/82	34.1%
Self	16/82	19.5%

Although nearly 20% of women participating on this study referred themselves and transferred their care from a different hospital to access OptiBreech Care, this likely underestimates the demand. Most sites reported difficulty facilitating transfers of care due to staff shortages, with some sites unable to accept late bookings for care. During the study period, the chief investigator received over 30 direct e-mail requests and phone calls from women pregnant at term with a breech baby, via the study’s website, asking for support and guidance because they could not access this at their local booking hospital. Most of these were not able to be accommodated in the study, due to severe staffing shortages throughout the pandemic or geographic distance.

#### Recruitment rate

The thirteen sites recruited 82 participants over 17 months. The per site overall mean recruitment was 0.71 (99% *CI* 0.40–1.02) participants per month. Higher recruitment rates occurred in centres with breech clinics and/or specialist midwives. The most reliable estimate of different effect size was seen in centres that had a visible breech specialist [35, 40] whether they were formally appointed in this role or informally fulfilling the same role, Table 5. Nine women changed their mind and planned a pre-labour caesarean birth after recruitment, for an attrition rate of 10.9%.

**Table5:** Recruitment in specialist vs non-specialist centres

	Number of sites	Total women recruited	Mean recruitment per month	Std. deviation	Std. error	95% confidence interval for mean	
						Lower bound	Upper bound
Breech specialist centres	8	66	0.90	0.310	0.110	0.64	1.16
No visible breech specialist	5	16	0.40	0.224	0.100	0.12	0.68
Total	13	82	0.71	0.369	0.102	0.49	0.93

Based on these recruitment data, to achieve optimal recruitment of approximately one woman per month who continues with a plan for a VBB, sites will need to be supported to host a dedicated clinic, with a breech specialist midwife able to co-ordinate the care pathway and disseminate training within the sites.

#### Implementation feasibility

A professional who had completed the OptiBreech training attended 87.5% (35/40, 95% *CI* 0.732–0.958) of VBBs in the study. A professional meeting the full proficiency criteria attended 67.5% (27/40, 95% *CI* 0.509–0.814) of VBBs. We only evaluated this criterion among actual VBBs because the OptiBreech team member’s role was to support the staff caring for the woman to acquire VBB skills safely. Once a decision was made for caesarean birth, there was often no need for the OptiBreech team to remain involved.

Our goal was to achieve 90% of vaginal births attended by a professional who had completed enhanced OptiBreech training, regardless of their experience level attending live breech births or managing complications in real time. We achieved 87.5% (35/40) overall in the study. One of the cases involving no OptiBreech attendant was a precipitous birth occurring at the woman’s home with no one in attendance, and one involved a rapidly progressing birth where the OptiBreech attendant was called but did not arrive in time for the birth. In another rapidly progressing birth, where the woman arrived at hospital fully dilated, a proficient OptiBreech attendant was present, but only for the last 20 min of the birth, rather than all of second stage as per the protocol.

The other two cases occurred at sites where ‘upright breech’ had been fully incorporated into mandatory training for some time. In one, no OptiBreech trained attendant was called because the consultant obstetrician felt confident with upright breech birth and facilitated the birth. At the other, no attempt was made to call someone with enhanced training, but the birth was managed by a midwife who felt confident with upright breech birth. Following discussion with the PI, the decision was made that the site did not have capacity to release staff for training. They therefore did not feel it was feasible to attempt to achieve this and withdrew from the study. The root causes in these two cases are modifiable.

Although the prespecified 90% target was not reached, following discussion with the SSC, it was decided that in 3/5 cases this was out of anyone’s control due to the unpredictable nature of birth, rather than unavailability of trained attendants or noncompliance. Rather than preventing a substantive study, this should be subject to further monitoring and informed consent.

#### Fidelity to physiological breech birth practice

Fidelity criteria were based on prior research concerning the principles of physiological breech birth [22] and elements included in the OptiBreech training package [24, 36]. No target was set, but instances where the fidelity criteria were not met were all investigated to understand what support sites might need to achieve maximum fidelity. Although the study was not powered to detect statistically significant differences, in all fidelity categories, greater fidelity to the protocol was observed with trained and/or proficient attendants at the birth (Table 6: Fidelity criteria).

**Table 6:** Fidelity criteria

	Attendant with OptiBreech training	None present with OptiBreech training	Attendant who met proficiency criteria	No one present meeting proficiency criteria	Total sample *n* (%)
**Maternal birth position**					
Upright	28 (80)	2 (50)	22 (81.5)	8 (66.7)	30 (76.9)
Supine	7 (20)	2 (50)	5 (18.5)	4 (33.3)	9 (23.0)
**Encouraged movement & effort**					
None required	6 (17.1)	1 (25.0)	5 (18.5)	2 (16.7)	7 (17.9)
Yes	25 (71.4)	2 (50.0)	21 (77.8)	6 (50.0)	27 (67.5)
No	4 (11.4)	1 (25.0)	1 (3.7)	4 (33.3)	5 (12.5)
** < 5-min pelvis to birth**					
Yes	31 (88.6)	3 (75.0)	24 (88.9)	10 (83.3)	34 (87.2)
No	4 (11.4)	1 (25.0)	3 (11.1)	2 (16.7)	5 (12.8)
**Total**	35/39 (89.7)	4/39 (10.2)	27/39 (69.2)	12/39 (30.7)	39^a^

^a^One birth before arrival/unassisted birth excluded. Where encouragement of movement and effort was not required, it meant that the birth occurred spontaneously, without direction from the attendant and without hands-on assistance

When women were attended by professionals who had not completed enhanced training, they used upright positions approximately 50% of the time. In each case, these professionals had exposure to the techniques through their annual mandatory training activities. When professionals had enhanced training and/or proficiency, this figure rose to 80% and above [43].

#### Maternal movement and effort

In approximately 1 in 5 births, neither verbal nor manual intervention was required for the birth to occur within the recommended time frame with good neonatal outcomes. Among proficient attendants, maternal movement and effort were used prior to hands-on assistance in 95% of cases. Among attendants who had completed OptiBreech training, fidelity to this criterion was 86%, and where no attendant completing OptiBreech training was present, it was 66%.

#### *Pelvis-to-birth interval* < *5 min*

This criterion was met 88.6% and 88.9% of the time by OptiBreech-training and proficient attendants, respectively, and 75% in births where neither were present.

#### Rumping-to-birth interval < 7 min

In March 2022, ‘rumping-to-birth interval < 7 min’ was added as a fidelity criteria due to a SAE in August 2021 and publication of research indicating this interval may be more important than the pelvis-to-birth interval [23]. Although this interval has always been part of the Physiological Breech Birth Algorithm [24], we added it as a fidelity criteria to emphasise its importance. Because of the short period between beginning to collect this information and the close of OptiBreech 1 (March 2022–June 2022), we only collected data on eight births. This interval was only exceeded in one of them, which was the only neonatal admission in this group. In this birth, the legs were assisted 1 min after the birth of the pelvis, but the arms were not born for another 3 min and are recorded as born spontaneously, assistance ‘not needed’. Assistance to deliver the head was initiated at 5 min after the pelvis was born, and the process took 4 min.

#### Resuscitation with umbilical cord intact

In March 2022, ‘initiation of resuscitation with the umbilical cord intact’ was also added as one of the fidelity criteria, due to feedback from participants, our PPIE group [41], and anecdotal reports from staff that this aspect of the training was not being achieved in practice. We were able to collect data on eight instances of resuscitation measures following VBBs. Among babies where neonatal admissions did not occur, three received resuscitative measures, but the team did not achieve stabilisation on the cord. In each of these instances, occurring in hospital settings, the OptiBreech team member felt resuscitation was not required, but other members of the team requested that the cord be clamped and cut so that the baby could be examined on the resuscitaire. In one instance, the foetal heart was confirmed as over 60 and improving, but the paediatric team preferred to examine the baby on the resuscitaire, so the umbilicus was clamped and cut prior to stabilisation, outside of guidance and the study protocol.

In each of these occurrences, OptiBreech team members felt that pressure from other members of the team contributed to inability to achieve this recommendation, as well as the physical environment, in which bedside resuscitation units are not standard and instead are attached to the wall or are too large to fit into the delivery rooms. One site does have a bedside stabilisation/resus unit, but it is ‘owned’ by the neonatal team and not able to be used outside of theatre, where it is reserved for preterm babies.

In the two other instances which did not involve neonatal admissions, despite 1-min Apgar score < 4, resuscitation was initiated with the cord intact, and the cord was not severed until after the onset of respirations. In one case, the environment was a home birth, where resuscitation stations are set up on the floor beside the birthing person as a matter of necessity. The other took place in the single participating site in which bedside resuscitation units (LifeStart™, Inspiration Healthcare, Crawley, UK) are used as standard.

In the three instances associated with neonatal admission, the cord was clamped and cut immediately. There is no record that the foetal heartrate was assessed prior to taking this action. Further implementation work is needed to be done to ensure fidelity to this aspect of the protocol.

#### Costs

Trained and/or proficient attendants spent a mean of 3.38 days and 6.49 nights on call to attend births in the study, Table 7.

**Table 7:** Time spent on-call

	**Number of births**	**Range (min–max)**	**Mean**	**Std. dev**
Total days on-call	78	0–16	3.38	4.267
Total nights on-call	78	0–29	6.49	7.632

When midwives work in on-call arrangements, for example in home birth teams, they are commonly paid a reduced rate for time spent on-call and an enhanced hourly rate if they are called in. At one site, the on-call rate is £1.49 per hour and time and a half when called in. However, breech specialists attend births to support the birthing person and their attending care providers, rather than to provide all clinical care. In this feasibility study, only one member of staff reported receiving payment for time spent on call, which was already part of her role within a community team. At most sites, being on call for breech births was voluntary, e.g. staff volunteered to attend if they were available, and/or was seen as a training opportunity for an extended role. All members of staff reported receiving back time in lieu or payment via staff bank arrangements if they had attended a VBB outside of their scheduled working hours.

In Table 8, costs associated with achieving breech proficiency through a breech clinic rotation [42], the estimated potential costs of training one senior midwife (Band 7) member of the team with minimal prior experience to full proficiency, are described [31]. This is estimated to be £10,213.

**Table 8:** Costs associated with achieving breech proficiency through a breech clinic rotation

Training to undertake presentation ultrasound scans				
	Hours	Cost (GBP)	Total (GBP)	Comment
Online or half-day theory course	3.5	62	217	
Ten supervised scans	3.5	62	217	Half-day in clinic with breech specialist
Cost of online training course			100	
Cost of acquiring proficiency with counselling/birth planning				
Observation	3.5	62	217	Half-day shadowing in clinic
Supervised practice	3.5	62	217	Half-day in clinic. Trainee continues to have access to breech specialist midwife and lead obstetrician for support
Training to acquire proficiency in facilitating physiological breech births				
On-line theoretical training	9.5	62	589	Current length of physiological breech birth online training package
Hands-on training day	7.5	62	465	Most people need in-person training to practice manoeuvres and for repetition required for adult learning
Supervised births (10 × 8 h)*	80	62	4960	Based on 5 h at birth + 1 h travelling + 2 h spent on-call
Cost of training package			100	Current cost of physiological breech birth in-person and on-line training package
Time spent delivering training	8	62	496	Delivering 2 h of mandatory training activities once per quarter
Additional counselling & support from specialist	48.5	62	2635	Based on 30-min additional counselling and 8 h of birth attendance, for 5/10 births
Total			£10,213	

Due to severe staffing shortages throughout the pandemic, sites were not able to support multiple people to be on-call. Therefore, other members of the team acquired experience with support of more experienced professionals on an ad hoc basis. Because of this, proficiency levels throughout the rest of the team developed more slowly than anticipated. In September 2022, one of the lead sites reported the first additional team member reaching proficiency, 1 year and 9 months after the start of OptiBreech 1. Therefore, our cost projections are hypothetical, based on the NHS reference costs of a Band 7 midwife and an estimated 8 h spent at each birth, agreed following discussion with PIs at the lead sites.

Tables 8, includes the enhanced physiological breech birth training package provided to OptiBreech team members. All NHS birth professionals attend mandatory annual training, which includes a brief VBB update, and this is not included. Our estimation also includes attendance at the 10 births to achieve proficiency and time for a currently proficient team member to attend to provide support to the trainee. This equates to an estimated £1021.13 per planned VBB, assuming the service only has one fully proficient attendant. Where some of the required competencies are already acquired, or less on-call time is needed, the cost required to achieve proficiency will be less.

#### Safety

The purpose of reporting safety outcomes in a feasibility study is to identify potential safety risks that would prevent a substantive study. The OptiBreech 1 study collected data on SAEs, neonatal admissions directly following birth and neonatal deaths only to ensure there was no obvious safety concern. All SAEs and neonatal admissions were reviewed with the SSC.

Four neonatal admissions occurred immediately following birth (4/82, 4.9%), one serious adverse outcome (1/82, 1.2%), and no neonatal deaths. The neonatal admission rate among actual vaginal breech births was 7.5% (3/40). We attempted to assess the background rate in participating sites during the 2 years prior to opening in OptiBreech 1; this was specified in the original protocol to assess the baseline safety. However, sites found this information difficult to collect retrospectively, and only five sites were able to provide it. In these five sites, 61 VBBs occurred during the 2 years prior to their opening in OptiBreech 1. These births had a neonatal admission rate of 13% (8/61), including one neonatal death.

One of the four neonatal admissions in OptiBreech 1 followed an in-labour caesarean after a bradycardia, with a breech presentation undiagnosed antenatally but vaginal birth attempted. No resuscitation was required, but the infant was admitted with suspected sepsis, and the mother was a known GBS carrier.

Two neonatal admissions occurred following prolonged second stages, which also involved circumstantial factors; in one case, an operating theatre was not immediately available. In both cases, there was disagreement within the team as to whether caesarean birth was indicated, and in the most serious case with the woman herself, who requested a caesarean in the second stage of labour. Following discussion with the SSC, further guidance around second-stage decision-making was provided to teams, and this has been incorporated into the OptiBreech Care Trial’s clinical guidance [28]. Sites were also encouraged to honour any woman’s request for a caesarean in labour, while recognising that sometimes this is not possible.

The fourth neonatal admission occurred following delay during emergence of the baby. The attendant had completed physiological breech birth training, but the site had not yet appointed a breech lead midwife. Following discussions with the CI and the SSC, the site was paused to recruitment until further face-to-face training could occur. Continual learning through discussion with sites contributed to on-going refinement of the OptiBreech training to support sites’ needs.

#### Effectiveness

We collected basic information on demographics and potential outcomes for a substantive study. Our cohort was evenly distributed between nulliparous and multiparous participants. Most participants were diagnosed prior to labour and had a previous failed attempt at ECV (Table 9).

**Table 9:** OptiBreech 1 basic demographics

	Nulliparous	Multiparous
Parity	41/82 (50.0%)	41/82 (50.0%)
	Yes	No
Diagnosed prior to labour	71/82 (86.6%)	11/82 (13.4%)
Had an attempt at ECV^a^	56/71 (78.9%)	15/71 (21.1%)

^a^Antenatal diagnosis only (71/82)

Tables 10, 11 and 12 present the mode of birth outcomes following intended VBB in the OptiBreech 1 study. This information is reported to facilitate counselling and informed choice for future research participants and future research estimated event rates. Cephalic births after spontaneous version are included because that is one of the outcomes of intention to treat by awaiting spontaneous labour. Outcomes are reported as a total sample (82), as a sample with those who changed their minds and requested a pre-labour caesarean removed (72) and as a sample with all pre-labour caesarean births removed (66). Our sample corresponds to the current RCOG guidance that, when a vaginal breech birth is planned, a CB will be performed approximately 40% of the time. Approximately 32% of the time, this will be in labour. These results will help to inform counselling in future research.

**Table 10:** OptiBreech 1 mode of birth outcomes

	**Total sample (%)**	**Total w/o maternal request planned CB**	**Total w/o planned CB**
Vaginal breech birth	38 (46.3)	38 (52.1)	38 (57.6)
Forceps breech	2 (2.4)	2 (2.7)	2 (3.0)
Cephalic birth	3 (3.7)	3 (4.1)	3 (4.5)
**Total vaginal births**	**43 (52.4)**	**43 (58.9)**	**43 (65.2)**
In-labour CB	23 (28.0)	23 (31.5)	23 (34.8)
Planned CB	16 (19.5)	7 (9.6)	-
**Total CB**	**39 (47.5)**	**30 (41.0)**	**23 (34.8%)**
Total	82	73	66

*CB* Caesarean birth

**Table 11:** Mode of birth stratified by parity and ECV attempt

	Nulliparous	Multiparous	Attempted ECV	No ECV attempt
Vaginal breech birth	14 (34.1)	24 (60.0)	23 (41.1)	15 (57.7)
Forceps breech	2 (4.9)	0	1 (1.8)	1 (3.8)
Cephalic birth	0	3 (7.5)	3 (5.4)	0
**Total vaginal births**	**16 (39.0)**	**27 (67.5)**	**27 (48.2)**	**16 (61.5)**
In-labour CB	14 (34.1)	9 (22.0)	17 (30.4)	6 (23.1)
**Total vaginal births after labour**	**16/30 (53.3)**	**27/36 (75.0)**	**27/44 (61.4)**	**16/22 (72.7)**
Planned CB	11 (26.8)	5 (12.2)	12 (21.4)	4 (15.4)
**Total CB**	**25 (60.9)**	**14 (34.2)**	**29 (51.8)**	**10 (38.5)**
Total	41	41	56	26

**Table 12:** Overall indications for caesarean birth

Reason	Number/39 (%)	Pre-labour CB/16 (%)	In-labour CB/23 (%)
Delay in labour	12 (30.8)		12 (52.2)
Changed mind/maternal request	9 (23.1)	9 (56.3)	
Maternal indication for delivery	4 (10.3)	3 (18.8)	1 (4.3)
Footling presentation	4 (10.3)	1 (6.3)	3 (13.0)
High estimated foetal weight	3 (7.7)	1 (6.3)	2 (8.7)
Non-reassuring foetal condition	2 (5.1)	1 (6.3)	1 (4.3)
Oligohydramnios	2 (5.1)	1 (6.3)	1 (4.3)
Cord prolapse	1 (2.6)		1 (4.3)
Advised against by non-OptiBreech professional	1 (2.6)		1 (4.3)
Premature rupture of membranes	1 (2.6)		1 (4.3)

Mode of birth is stratified below by parity and whether the person had a prior failed attempt at ECV, for the same purposes. In OptiBreech 1, multiparous and women who had no previous attempt at ECV gave birth vaginally more often than nulliparous women and those who did have a failed ECV attempt.

Indications for caesarean birth are reported as a percentage of the overall total of caesarean births that occurred in this cohort. This is to identify whether any indications were potentially modifiable in future research to improve outcomes for women while maintaining safety for babies.

## Discussion

The OptiBreech 1 study demonstrated that, despite minimal experience levels at the start of the study, it is feasible to provide team care for planned physiological breech births most of the time. Demand for the service was higher than expected, with one in five participants transferring care from an outside hospital to access OptiBreech care. This suggests that sufficient demand exists to support a larger study, and that the proposed care pathway is meeting a need that is not consistently met within current NHS services. This resonates with the consistent feedback we have received from our PPIE group.

The OptiBreech 1 study showed no indication not to proceed with a pilot trial on safety grounds, as the available prospectively collected data suggest a possible improvement in outcomes. Future research should aim to collect data on all breech births occurring within OptiBreech settings. Although neonatal admission is a poor proxy for serious and long-term outcomes, it has economic implications and has been associated with poorer long-term outcomes following vaginal breech births [43]. The only other available data comes from the previous evaluation of the physiological breech birth training package used in the OptiBreech 1 study. In that study, among births where no one who had attended the training was present, the incidence of serious neonatal morbidity was 5/69 (7.2%), compared to 0/21 among births where someone who had completed the training was present [36].

For various reasons, approximately 12.5% of planned OptiBreech births may not be attended by fully OptiBreech-trained attendants. Any future studies should ensure women are informed of this possibility, and the impact of trained and/or proficient attendants on the outcomes of VBBs should continue to be observed. The full proficiency criteria include participation in clinical teaching [39]. Qualitative research demonstrates teaching contributes to consolidation of a specialist’s skill and knowledge while generating skill and knowledge among the wider clinical team and making the specialist a more visible leader within the service [35, 38]. Full implementation of OptiBreech care should include ensuring proficient team members deliver annual breech updates so that, in cases where an experienced team member is not available, care is still likely to be provided with fidelity to the protocol.

The finding that upright positioning was used increasingly following OptiBreech training and proficiency accords with previous physiological breech birth training evaluation data, in which this figure was also 80% following training [36]. Where professionals receive adequate training, both women and professionals appear to find upright birth positioning in vaginal breech births acceptable, and a majority prefer it.

Estimating substantive study recruitment based on feasibility figures is challenging [44]. Recruitment in feasibility studies is often higher than figures achieved in the substantive studies. One reason is that sites that participate in feasibility studies often have a greater interest in or commitment to testing the proposed intervention. Our experience indicates that recruitment of active sites is likely to be dependent on the presence of a champion within the service and support from senior management.

Vaginal breech birth has been a problematic and conflicted area of practice for some time [6], and PIs described being keen to help find a solution. This appeared to be what motivated clinical midwives and obstetricians to become involved in leading and delivering research for the first time. Not all were successful in their attempts. Managing resistance and conflict took a toll on several potential and actual PIs, due to frustrations at being blocked in their attempts to deliver the standard of care they wished to provide for women [45, 46]. While these highly motivated PIs made delivery of this research possible, supporting novice research leaders involved an investment in time and capacity building from the central research team; this needs to be adequately planned for in any future study.

Sites reported difficulty facilitating transfers of care due to staff shortages. Future studies may need to ensure participating sites receive the support they need to accept women transferring to access specialist care, as would occur if the model was scaled up.

There may also need to be specialist support for staff leading the service/research or affected by severe adverse outcomes. Despite the best efforts of clinical staff, severe adverse outcomes are occasionally unavoidable, regardless of the way people choose to give birth to their breech babies. When introducing new practices in an area already known to be at higher risk, adverse outcomes are likely to occur, and appropriate support should be available.

This study observed implementation of physiological breech birth support through provision of proficient team care. An alternative training and implementation strategy, providing enhanced training to a larger number of staff, has recently been evaluated in NHS settings [36]. In that evaluation, training 195 staff to attend a 1-day training was estimated to cost £62,434. In the year following training, 21/53 births were attended by professionals who had completed the training, and outcomes were better among these births than those not attended by someone who had not completed the enhanced training. This equates to a cost of £2973 per birth, compared to the £1021.13 per planned VBB estimated in the proficient team care model. In the previous evaluation, 20/21 of these occurred in sites that adopted a policy of calling professionals with enhanced training to the births wherever possible. In sites where this did not occur, providing enhanced training to significant numbers of staff resulted in minimal benefit. In services where overall skill levels have been depleted, targeted training and development of a small number of staff enabled to work flexibly [35], and calling them to VBBs wherever possible appears to be a more cost-effective initial implementation strategy. OptiBreech care is also highly acceptable to women planning a VBB [31].

## Conclusion

The results of the OptiBreech 1 feasibility study indicate that it is possible to implement OptiBreech team care for vaginal breech births to test the randomisation procedures in a pilot trial. This premise was tested during a pandemic, in contexts with low overall experience and proficiency levels. The model depends on the motivation of front-line clinicians to deliver the care innovation and the research and the support of their colleagues and management. These factors should be taken into consideration in future site selection. In addition, for approximately 1:10 births, an OptiBreech-trained attendant may not be available, due to the unpredictability of spontaneous labour. Women should be informed of this possibility, and future research should monitor this and its association with outcomes. The next stage of this research is continuation of the prospective observational cohort study with a nested pilot randomised trial comparing OptiBreech collaborative care with standard care in the four leading sites.

## Data Availability

The datasets generated and analysed during the OptiBreech 1 study are available in the Figshare repository, https://doi.org/10.6084/m9.figshare.c.6238653.v2.
